# Supplementary material for: Effects of Deferasirox in Alzheimer’s Disease and Tauopathy Animal Models
Source: Biomolecules. 2022 Feb 25;12(3):365. doi: 10.3390/biom12030365 (PMC8945800; doi:10.3390/biom12030365)
Supplement: Supplementary file 1 [file biomolecules-12-00365-s001.zip › biomolecules-1563641-supplementary.pdf]

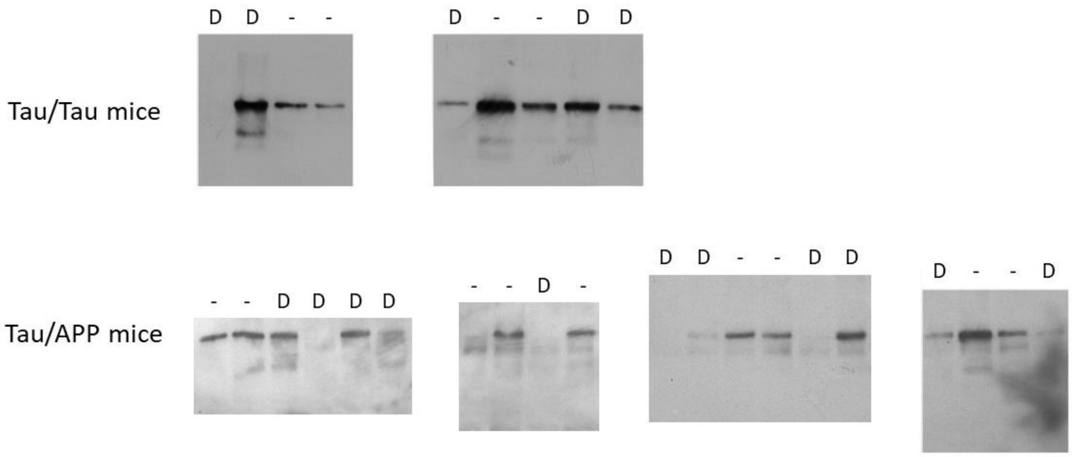

Figure S1: Western blots. Effect of deferasirox treatment as seen in Western blots of a brain fraction enriched in paired helical filaments (PHF) and subjected to AT8 immunostaining to measure accumulation of the 64 kD band of phosphorylated tau. D = deferasirox treated mouse, - = mouse not treated with deferasirox.
